# Editorial: Improving the gut microbiome: applications of fecal transplantation in disease, volume II

**DOI:** 10.3389/fmed.2025.1758943

**Published:** 2026-01-06

**Authors:** Angel Lanas, Ana-Isabel Alvarez-Mercado

**Affiliations:** 1 Aragón Health Research Institute (IIS Aragón), Centro de Investigación Biomédica en Red: Enfermedades Hepáticas y Digestivas (CIBERehd), University of Zaragoza, Zaragoza, Spain; 2 Instituto Biosanitario de Granada (Ibs GRANADA), University of Granada, Granada, Spain

**Keywords:** *Clostridioides difficile*, colonoscopy, dysbiosis, fecal transplantation, microbiome

The last decade has reshaped our understanding of the gut microbiota from a passive microbial community to a dynamic ecosystem essential to host physiology, immunity, metabolism, and clinical outcomes across multiple organ systems. Advances in high-throughput sequencing, metabolomics, and mechanistic microbiome science have revealed that dysbiosis is not only a biomarker of disease but can act as a causal driver of inflammatory, metabolic, immunologic, and neurobehavioral disorders. As a result, there is unprecedented interest in therapeutic strategies that restore or modulate the intestinal microbiota.

Fecal microbiota transplantation (FMT) has emerged as the most direct and potent method to reconstitute gut microbial communities, offering the ability to correct dysbiosis rapidly and comprehensively in ways not attainable by diet, probiotics, or prebiotics alone. While established as the most effective therapy for recurrent *Clostridioides difficile* infection, FMT is now being explored across a broad spectrum of diseases—from metabolic dysfunction and sepsis to intestinal inflammation, kidney injury, and even behavioral and neurological disorders. The breadth of potential applications reflects a paradigm shift: the microbiome is increasingly seen as a therapeutic target and interventional node rather than a static correlate of disease.

This second volume of the Research Topic “*Improving the gut microbiome: applications of fecal transplantation in disease*” highlights this expanding field. The five accepted articles span methodologic innovation, mechanistic experimentation, clinical synthesis, and global research mapping. Together, they illustrate the rapid evolution of FMT from a niche procedure to a versatile biological therapy with cross-disciplinary impact. Below, we summarize the major contributions of each manuscript and situate them within the broader direction of microbiome-based medicine.

Li et al. address a practical yet consequential challenge in clinical gastroenterology: achieving safe, reproducible placement of catheters for repeated lower-gastrointestinal FMT administrations. Their retrospective analysis evaluates two modified colonoscopically guided catheter placement methods—the Direct Loop Clamping technique and the Clip Loop Binding technique. Both demonstrated 100% placement success, with low adverse-event rates and comparable procedure times. Notably, the Clip Loop Binding method provided a shorter initial clip securement time, potentially offering workflow advantages. By simplifying technical steps, reducing material costs, and enabling rapid repeat instillations over multiple days, this work advances the operational feasibility of FMT. Such innovations are essential for expanding FMT availability beyond specialized centers and increasing its accessibility for patients requiring intensive or serial dosing protocols. The limitations of the study however limits generalizability and include the single-center retrospective design, an incomplete procedural documentation, absence of randomization, which prevents controlled comparison, and no assessment of downstream clinical outcomes.

In a compelling animal study, Han et al. explore the role of microbiota restoration in juvenile sepsis, a context where antibiotic exposure, immune dysregulation, and high mortality converge. Using a cecal ligation and puncture model, the authors show that antibiotic-induced dysbiosis markedly worsens inflammatory cytokine responses and reduces survival. Strikingly, FMT not only reverses dysbiosis but restores survival rates to those of healthy controls (85.7%), indicating powerful protective effects. These data underscore the emerging concept of the microbiota–immune axis in critical illness. They also raise important translational questions: Can early microbiota correction mitigate sepsis progression in pediatric intensive care? How might timing, donor selection, or route of administration influence outcomes? This study provides foundational evidence to justify future clinical exploration. Again, we need to point out a number of study limitations, since findings derive from a single animal model, the prophylactic design does not mirror real-world clinical timing and mechanistic exploration beyond cytokine dynamics is limited. Therefore, translation to pediatric sepsis requires caution.

Abildinova et al. present a comprehensive bibliometric analysis (2000–2024) of global scholarship linking the gut microbiota with insulin resistance. Their dataset of 1,884 studies reveals exponential growth, an expanding collaborative network, and a strong emphasis on mechanisms involving short-chain fatty acids, gut–hormone signaling, and inflammation. The authors identify leading institutions, high-impact articles, and shifting thematic priorities, offering a strategic overview for researchers entering this domain. Within the context of FMT, this bibliometric mapping highlights a key trend: metabolic diseases have become one of the fastest-growing areas of microbiome-targeted research. The findings suggest that the next decade will likely see more interventional studies—including FMT or microbial consortia—focused on obesity, diabetes, and metabolic syndrome. Nevertheless, bibliometric approaches cannot assess methodological rigor or reproducibility beyond the potential selection bias and which is more important, the descriptive nature of this type of studies precludes qualitative evaluation of scientific advances.

In an extensive mini-review, Cao et al. synthesize recent human-focused evidence on the benefits, risks, and therapeutic mechanisms of FMT. Their review spans metabolic disorders, intestinal diseases (including CDI, IBS, and IBD), neurobehavioral conditions, immune-mediated diseases, antimicrobial resistance, and emerging combination strategies (e.g., FMT with dietary modifications, selenium supplementation, or washed-microbiota preparations). They key highlights include: (a) the clinical improvement in obesity, NAFLD, and diabetes via microbial re-engraftment; (b) the durable benefit in CDI with emerging oral formulations and microbial ecosystem therapeutics; (c) the modulation of the gut–brain axis with symptom reduction in anxiety, depression, and autism spectrum disorders, and (d) the evidence that FMT reduces antimicrobial-resistance gene burden and multidrug-resistant organism colonization.

The authors also emphasize persistent challenges—variability in donor material, safety risks in immunocompromised populations, incomplete mechanistic understanding, and the need for standardized protocols. This review provides clinicians with an up-to-date, balanced appraisal of FMT's expanding clinical footprint. Within the limitations of the study we need to point out the narrative synthesis without a systematic methodology, the broad topic scope limits depth of mechanistic detail and the heterogeneity in included clinical studies makes cross-comparison challenging.

Finally, Yu et al. provide new mechanistic insights into the gut–kidney axis by demonstrating that prophylactic FMT alleviates ischemia–reperfusion injury in rats through propionic acid–mediated activation of GPR43 signaling. Their use of enteric-coated FMT capsules is particularly notable, as it eliminates gastric acid–related microbial loss and models a clinically scalable delivery method. The study shows that FMT improves renal function and histopathology after IRI, reduces systemic and intrarenal inflammatory cytokines, decreases tubular apoptosis and enhances proliferation, reshapes the microbiota with enrichment of Lachnospiraceae, a key SCFA-producing family, and elevates propionic acid levels that activate GPR43, suppressing NF-κB-mediated inflammation. By bridging microbial ecology, metabolomics, and receptor signaling, this work illuminates a precise biochemical pathway through which FMT confers systemic organ protection. It opens avenues for targeted microbial or metabolite-based therapeutics as adjuncts in acute kidney injury prevention. Among the limitations of the study, it can be said that prophylactic application limits somehow its clinical relevance, cross-species FMT complicates ecological interpretation, that mechanisms shown were not validated at microbial strain level and that long-term kidney outcomes were not studied.

Collectively, the articles in this Research Topic reinforce several central themes:

1. FMT is transitioning from empirical therapy to mechanism-guided intervention, supported by advances in sequencing, microbial metabolite profiling, and immunologic analysis.
2. Technical and methodological innovation—including optimized catheter placement techniques and enteric-coated capsules—is improving procedural safety, consistency, and accessibility.
3. Mechanistic studies are uncovering immunologic and metabolic pathways—from SCFA signaling to cytokine modulation—that underpin FMT's therapeutic effects across organ systems.
4. Applications of FMT continue to expand, now spanning critical care, metabolic health, nephrology, behavioral science, and infectious diseases.
5. Global research activity is rapidly accelerating, with rising interdisciplinary collaboration and an emerging focus on the microbiome as a modifiable determinant of disease.

Despite these advances, among others, key challenges remain: (a) establishing standardized FMT preparations, (b) ensuring long-term safety, (c) optimizing donor selection strategies, and (d) conducting adequately powered clinical trials across diverse patient populations. As the field advances toward precision-designed microbial therapies, these studies collectively move us closer to safe, mechanism-based, and personalized microbiome interventions capable of reshaping how we approach disease prevention and treatment in gastroenterology and beyond.

